# Epilepsy-associated *GRIN2A* mutations reduce NMDA receptor trafficking and agonist potency – molecular profiling and functional rescue

**DOI:** 10.1038/s41598-017-00115-w

**Published:** 2017-02-27

**Authors:** L. Addis, J. K. Virdee, L. R. Vidler, D. A. Collier, D. K. Pal, D. Ursu

**Affiliations:** 1 Department of Basic and Clinical Neuroscience, Institute of Psychiatry, Psychology and Neuroscience, King’s College London, London, UK; 2 Neuroscience Discovery, Eli Lilly Research Centre, Windlesham, Surrey UK

## Abstract

Mutations in the N-methyl-D-aspartate receptor (NMDAR) gene *GRIN2A* cause epilepsy-aphasia syndrome (EAS), a spectrum of epileptic, cognitive and language disorders. Using bioinformatic and patient data we shortlisted 10 diverse missense mutations for characterisation. We used high-throughput calcium-flux assays and patch clamp recordings of transiently transfected HEK-293 cells for electrophysiological characterization, and Western blotting and confocal imaging to assay expression and surface trafficking. Mutations P79R, C231Y, G483R and M705V caused a significant reduction in glutamate and glycine agonist potency, whilst D731N was non-responsive. These mutants, along with E714K, also showed significantly decreased total protein levels and trafficking to the cell surface, whilst C436R was not trafficked at all. Crucially this reduced surface expression did not cause the reduced agonist response. We were able to rescue the phenotype of P79R, C231Y, G483R and M705V after treatment with a GluN2A-selective positive allosteric modulator. With our methodology we were not able to identify any functional deficits in mutations I814T, D933N and N976S located between the glutamate-binding domain and C-terminus. We show *GRIN2A* mutations affect the expression and function of the receptor in different ways. Careful molecular profiling of patients will be essential for future effective personalised treatment options.

## Introduction

The epilepsy-aphasia spectrum (EAS) represents a continuum of genetic epilepsy syndromes with the EEG signature of focal sharp waves, concurrent with various speech and language disorders^1, 2^. At the mild end is the common childhood disorder of Rolandic epilepsy (RE), frequently associated with speech and oromotor deficits, as well as reading disability, attention, and memory problems^3^. Focal seizures commonly occur during sleep and the defining electroencephalographic (EEG) abnormality is blunt centrotemporal spikes (CTS) during sleep^4^. Atypical benign partial epilepsy (ABPE) is much more rare, consisting of more severe seizures as well as some regression of speech, motor skills and attention^5^. The debilitating disorders of epileptic encephalopathy of continuous spike-and-waves during slow-wave sleep (ECSWS) and Landau-Kleffner syndrome (LKS) represent the more severe, and less-frequent, end of the spectrum. There is catastrophic loss of receptive and expressive language in children who develop LKS and who were, in general, previously cognitively normal. The EEG evolves into continuous spike-and-waves during slow-wave sleep (CSWS)^6^, with easily controlled Rolandic seizures. In ECSWS, the epilepsy is often refractory to treatment, with multiple seizure types present. Children with ECSWS have global regression across learning, memory, motor and social skills^7^.

**Figure 1:**
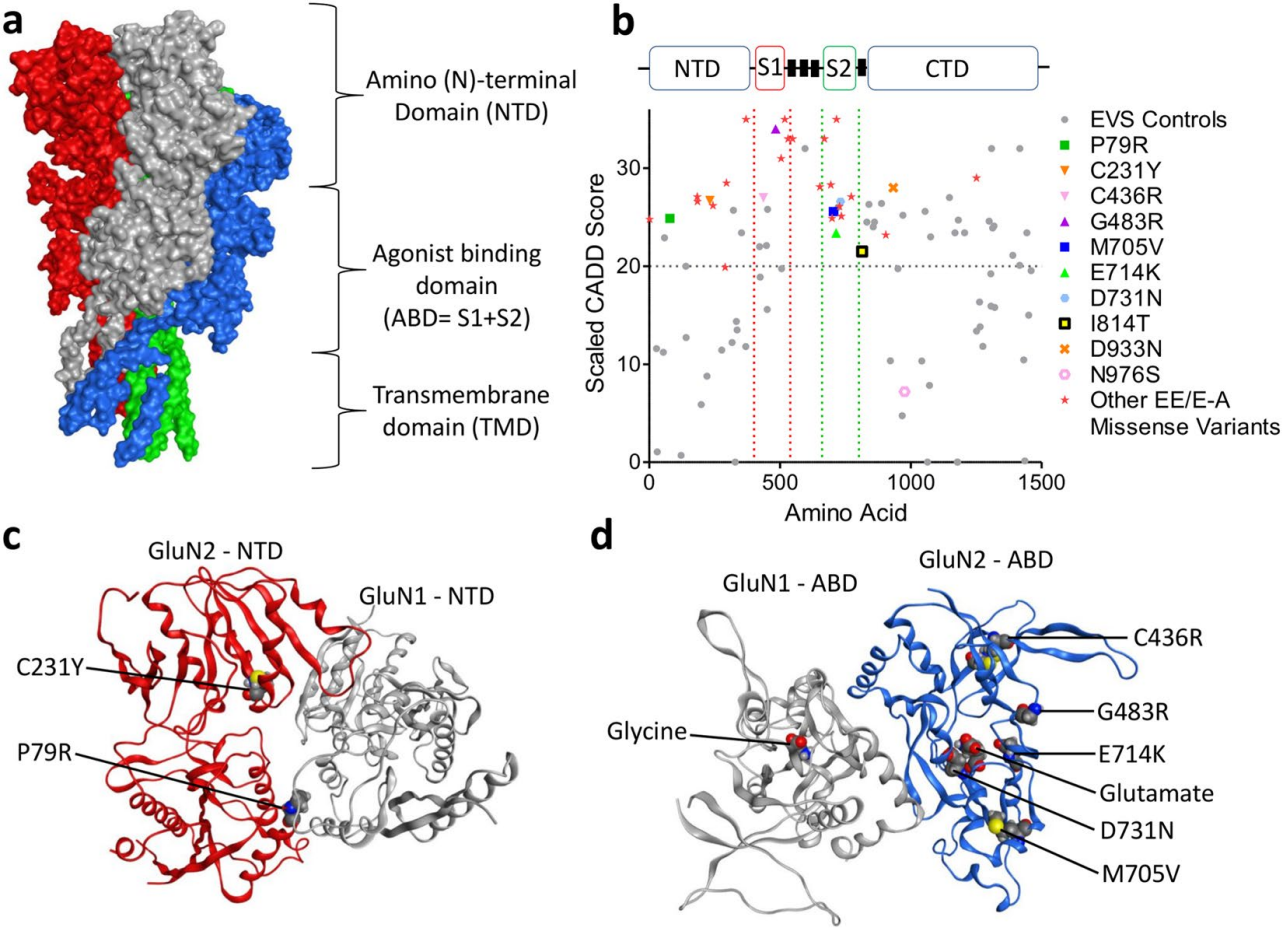
CADD scores and protein modelling predict stronger functional effects for EAS-associated *GRIN2A* mutations than in controls. (**a**) Protein structure model of NMDAR (PDB ID 4TLL): GluN1 (grey and green), GluN2 (B in this structure) (blue and red). Membrane would be horizontal in this image with the NTD and ABD in the extracellular space. Intracellular C-Terminal domain would be below the transmembrane domain (not present in this structure). (**b**) Schematic linear representation of GluN2A with the domains annotated. Black rectangles indicate transmembrane domains. Plot of scaled CADD scores against GluN2A amino acid position for missense variants. Black dots represent scores for 65/6474 individuals from the Exome Variant Server (EVS) that had missense variants in *GRIN2A* and coloured symbols are scores for variants found in individuals with EAS disorders. The horizontal dotted line indicates the scaled CADD score cut off of 20 for a highly likely deleterious variant. (**c**) Protein structure model of the NTD of NMDAR (PDB ID 3QEL): GluN1 (grey), GluN2 (B in this structure) (red). Mutations considered in this domain highlighted (conserved between GluN2A and B). (**d**) Protein structure model of the LBD of NMDAR (PDB ID 2A5T): GluN1 (grey), GluN2A (blue). Mutations considered in this paper highlighted, as well as agonists.

Until relatively recently the aetiology of most EAS disorders was largely unknown, although environmental factors such as thalamic injury can cause the EEG phenotype^8^. CTS in RE is associated with variants in *PAX6-ELP4* on 11p13^9, 10^, and rare mutations and deletions in the N-methyl-D-aspartate receptor (NMDAR) genes have been reported in families with mental retardation and various epilepsy phenotypes^11–14^. In 2013 three groups simultaneously discovered mutations and small deletions of *GRIN2A* across EAS disorders^15–17^. *GRIN2A* encodes the GluN2A subunit of the NMDAR, a transmembrane ligand-gated ion channel with a critical role in normal neuronal development, synaptic plasticity and memory. Disturbances of NMDARs are implicated in disorders such as schizophrenia and Alzheimer’s disease and represent targets of interest for pharmacological treatments^18, 19^. NMDARs are hetero-tetramers, usually consisting of two GluN1 and two GluN2 (A-D) subunits, creating many receptor subtypes with distinct properties, functions and expression levels^14, 19^. NMDARs are activated following the binding of glycine and glutamate to the GluN1 and GluN2 subunits respectively. This then opens the cation-selective pore, allowing intracellular Ca^2+^ influx when the Mg^2+^ blockade is removed upon membrane depolarisation. NMDARs consist of four domains: the extracellular amino (N)-terminal domain (NTD) involved in subunit assembly and allosteric modulation, (Fig. 1a), the agonist-binding domain (ABD), the transmembrane domain (TMD), and the intracellular C-terminal domain (CTD) involved in receptor trafficking^14^.

Here we present an *in vitro* functional analysis of 10 missense mutations of *GRIN2A* identified in children with EAS disorders^15–17^. Our studies reveal variable phenotypes ranging from loss of receptor trafficking, through altered agonist potency to total loss of function. We were able to rescue the phenotype of mutations with reduced glutamate potency following treatment with a GluN2A-selective positive allosteric modulator. This work indicates careful molecular analysis of *GRIN2A* patient mutations is important before starting targeted treatment options.

## Results

### Bioinformatic analysis and selection of *GRIN2A* patient mutations

Over 30 *GRIN2A* missense mutations have been identified in EAS patients^15–17^. We assigned Combined Annotation Dependent Depletion (CADD) scores^20^ to these mutations to create predictions of deleteriousness based on measures such as conservation metrics and protein structure. These CADD scores were compared to those for controls from the Exome Variant Server (EVS), (Fig. 1b). All EAS-associated variants apart from N976S are located above the CADD-20 cut off for the 1% most deleterious variants, and 26/31 are located within the NTD, ABD or pore domains. Variants from EVS controls either largely score below CADD-20 or are located within the C-terminus where they are predicted to cause less severe functional effects.

We selected 10 EAS-associated missense mutations for further analysis from different functional domains, with different inheritance patterns (*de novo* vs inherited and segregating with disease), disease outcomes and a range of CADD scores (Table 1 and Fig. 1). A contribution to the selection of the mutants was hypothesis driven, based on analysis of crystal structures, (Fig. 1c).

**Table 1:** Summary of clinical and genetic information for studied *GRIN2A* mutations.

cDNA alteration	Protein alteration	Phenotype	Inheritance, Segregation (Y/N)	CADD Scaled Score	Reference
c.236C > G	P79R	RE/ECSWS	Maternal, Y	24.9	16
c.692G > A	C231Y	LKS	Maternal, Y	26.7	16
c.1306T > C	C436R	ABPE	*de novo*	27	16
c.1447G > A	G483R	ECSWS, aRE	Maternal, N	34	17
c.2113A > G	M705V	RE	Maternal, Y	25.6	16
c.2140G > A	E714K	ECSWS	Unknown	23.4	16
c.2191G > A	D731N	aRE, VD	Maternal, VD -Y	26.6	17
c.2441T > C	I814T	RE	Paternal, N	21.5	16
c.2797G > A	D933N	LKS	Paternal, N	28	17
c.2927A > G	N976S	ECSWS	Unknown	7.2	16
		ABPE	Unknown		

cDNA annotation based on the canonical transcript ENST00000396573. Phenotype abbreviations as follows: ABPE – atypical benign partial epilepsy, aRE – atypical Rolandic epilepsy, CSWS – epileptic encephalopathy with continuous spike and wave during slow wave sleep, LKS – Landau-Kleffner Syndrome, RE – Rolandic epilepsy, VD – verbal dyspraxia. Y-yes, N-no for segregation of variants with phenotype. CADD score stands for “combined annotation dependent depletion”, a tool for scoring the deleteriousness of single nucleotide variants and were generated using v1.3 on 19 May 2016 from http://cadd.gs.washington.edu/. A score > 20 indicates the top 1% most deleterious substitutions^20^.

### Selected *GRIN2A* mutations protect against NMDA receptor toxicity

We carried out site-directed mutagenesis in the human *GRIN2A* cDNA to create the 10 mutation constructs, which were subsequently inserted into the expression plasmid pcDNA3.1(+) before transfection into HEK-293 cells along with *GRIN1* in a second pcDNA3.1(+) plasmid. To allow identification of the transfected cells an HA-tag was added to the N-terminus, which did not alter the function of the receptor (Supplementary Fig. 1) and this construct was subsequently called Wild Type (WT).

**Figure 2:**
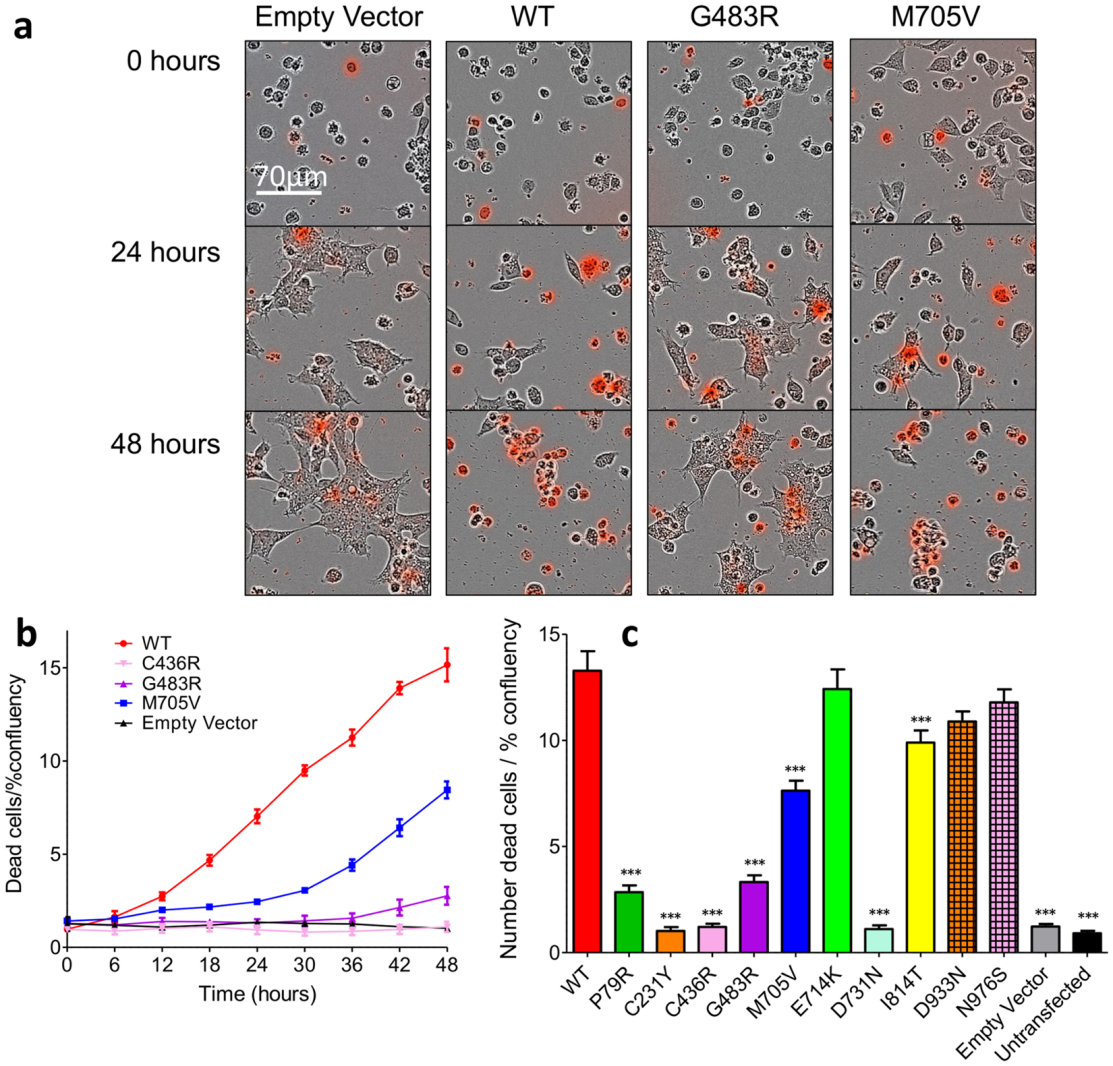
Selected *GRIN2A* mutations protect against glutamate-induced toxicity in HEK cells. (**a**) Superimposed bright field and pseudocolour red images, indicating fluorescent Cytotox Red dye in dead cells, of HEK cells transiently transfected with various *GRIN2A* mutant constructs, or empty vector. Free glutamate and glycine in the culture media causes cell death in those expressing functional NMDARs over 48 hours. Images captures at 20x using the IncuCyte live-cell imaging system. (**b**) Representative time course of cell death, normalised to initial confluency in each well, n = 5 wells, Mean ± SEM. (**c**) Plot of cell mortality for *GRIN2A* mutants normalised to initial confluency per well. Mutants P79R, C231Y, C436R, G483R, M705V, D731N and I814T are protective against glutamate toxicity either due to reduced trafficking and/or reduced functionality of the receptors. ***p < 0.001 Dunnett’s corrected one-way ANOVA. Averaged data from n = 15 wells per construct except for G483R, E714K, D933N and N976S where n = 12 wells, 3 × 10^4^ cells/well. Mean ± SEM.

Free glutamate and glycine in culture media is toxic to HEK cells transfected with NMDAR from direct influx of calcium through the activated channels^21^. Using a continuous live-cell imaging system we were able to leverage this characteristic to carry out a screen for GluN2A mutant functionality. After 48 hours almost all of the WT GluN2A cells were dead, compared to very few of those co-transfected with the control empty vector and *GRIN1*, which were spread out and dividing. Some mutants showed an intermediate phenotype (Fig. 2a). Cells transfected with the WT *GRIN2A* receptor die at an accelerated rate compared to those that are untransfected (UT) or transfected with an empty vector after only 12 hours (Fig. 2b). This indicates the rapid expression and trafficking of the NMDAR to the cell surface, the binding of glutamate present in the media and the subsequent calcium influx and excitotoxicity. Analysis of the GluN2A mutants shows a similar mortality rate to WT for E714K, D933N and N976S indicating similar functionality, (Fig. 2c). All other mutants were significantly different to the WT construct (Dunnett’s corrected AVOVA, p < 0.001). I814T and M705V appear to have an intermediate phenotype, followed by G483R and P79R with less than 75% of cells dying after 48 hours. A level of mortality consistent with cells transfected with the empty vector is shown for C231Y, C436R and D731N.

### *GRIN2A* mutations can decrease agonist potency

**Figure 3:**
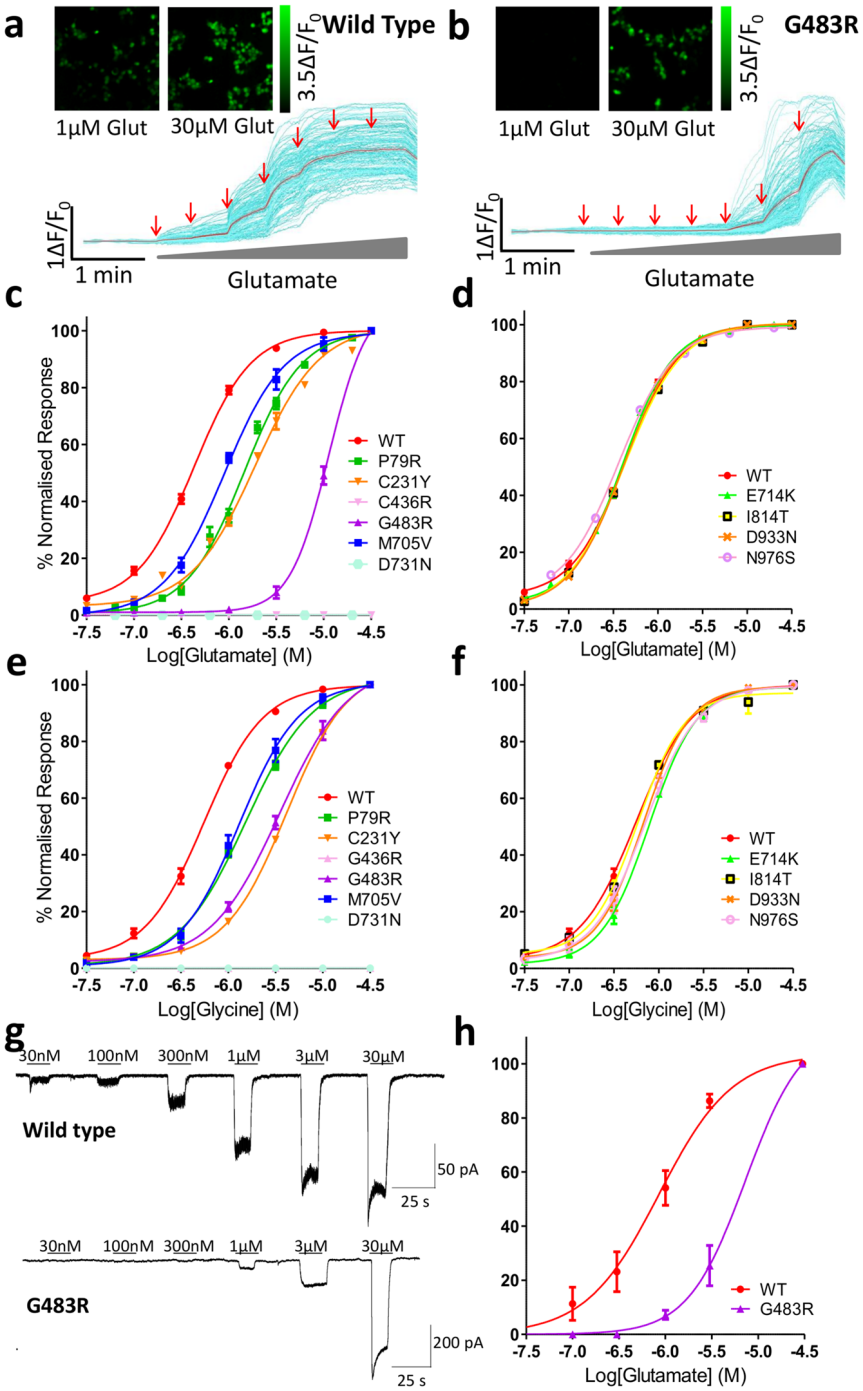
*GRIN2A* mutations alter the response to glutamate and glycine. (**a,b**) Pseudocolour images and representative single-cell traces from calcium-flux imaging for HEK cells transfected with either WT or G483R *GRIN2A* showing different calcium responses caused by the application of increasing concentrations of glutamate (30 nM–30 µM red arrows). Individual cell traces in cyan and the mean response in red. (**c**,**d**) Normalised concentration response curves (CRCs) to increasing concentrations of glutamate from single cell calcium-flux imaging. In (**c**) 4 mutants; P79R, C231Y, C483R and M705V have reduced agonist potency, while C436R and D731N show no response to glutamate. Four mutant constructs (**d**), show an unaltered response to glutamate. (**e,f**) Normalised CRCs to increasing glycine concentration with constant 3 µM glutamate – the mutations show similar responses as to glutamate. See Table 2 for n to create averaged data per construct, 3 × 10^4^ cells/well. Error bars ± SEM. (**g**) Representative continuous voltage-clamp recordings obtained from HEK cells transfected with WT or G483R *GRIN2A* plasmid. Bars above the recording indicate glutamate application (co-applied with 30 µM glycine). Application of increasing concentrations of glutamate shows a progressive increase in current observed, the sensitivity of which is shifted to higher concentrations of glutamate in the G483R mutant compared to WT. (**h**) Normalised CRC to glutamate as recorded in (**g**) indicates the same response as for single cell calcium imaging (n = 3).

The results of the cell toxicity assay indicated that some mutants potentially alter GluN2A glutamate potency and/or receptor expression. We used fluorescence-based single-cell calcium-flux imaging to investigate agonist pharmacology in more detail in transiently transfected HEK cells^22, 23^. Four mutants, P79R, C231Y, C483R and M705V significantly decrease glutamate potency (F_1,63_ = 459.1, F_1,55_ = 427.3, F_1,43_ = 987.6 and F_1,43_ = 160.9 respectively, Bonferroni corrected ANOVA, p < 0.0001), (Fig. 3a–c), as shown by the increase in the half-maximally effective concentration of agonist (EC_50_) (Table 2). Two mutations, C436R and D731N produced no detectable response to maximal glutamate or glycine, (Fig. 3c,e). Four of the mutations did not alter the response to glutamate, E714K, I814T, D933N and N976S, when compared to WT, (Fig. 3d). The effects on glycine potency of the *GRIN2A* mutations were similar to those recorded for glutamate with P79R, C231Y, C483R and M705V producing significant decreases in glycine potency compared to WT receptors (F_1,36_ = 246.2, 539.7, 347.2 and 116.3 respectively, p < 0.0001), (Fig. 3e,f and Table 2). These shifts in glutamate and glycine potency are not due to saturation of the Fluo-4AM calcium dye, as use of the higher-affinity Fluo-2AM did not alter the CRC curves (data not shown). Single cell patch clamp recordings of HEK cells transfected with G483R-*GRIN2A* and *GRIN1* gave very similar results to the calcium-flux imaging, (Fig. 3g,h).

**Table 2:** Summary of Glutamate and Glycine EC_50_ for wild type and mutant GluN2A constructs.

Mutation	Glutamate EC_50 _µM, 95%CI (n)	Glycine EC_50 _µM, 95%CI (n)
**WT**	0.42, 0.40–0.47 (16)	0.55, 0.50–0.61 (12)
**P79R**	1.45, 1.35–1.57 (15)***	1.53, 1.37–1.70 (11)***
**C231Y**	1.89, 1.71–2.10 (17)***	4.13, 3.86–4.42 (13)***
**C436R**	—	—
**G483R**	11.30, 10.1–12.65 (12)***	3.50, 3.01–4.08 (12)***
**M705V**	0.89, 0.78–1.02 (12)***	1.31, 1.11–1.53 (11)***
**E714K**	0.41, 0.38–0.45 (12)	0.78, 0.72–0.85 (13)
**D731N**		—
**I814T**	0.42, 0.38–0.44 (9)	0.58, 0.50–0.67 (10)
**D933N**	0.41, 0.38–0.44 (9)	0.68, 0.62–0.75 (10)
**N976S**	0.37, 0.32–0.43 (9)	0.71, 0.60–0.83 (11)

Data expressed as mean (95% confidence intervals). N = number of wells from the single cell calcium imaging assays (3–6 assays). When no values are given this is because there was no detectable response. ***P < 0.0001 Bonferroni corrected extra-sum-of-squares F-test of best-fit values of LogEC_50_.

### *GRIN2A* mutations can alter total levels of GluN2A protein

**Figure 4:**
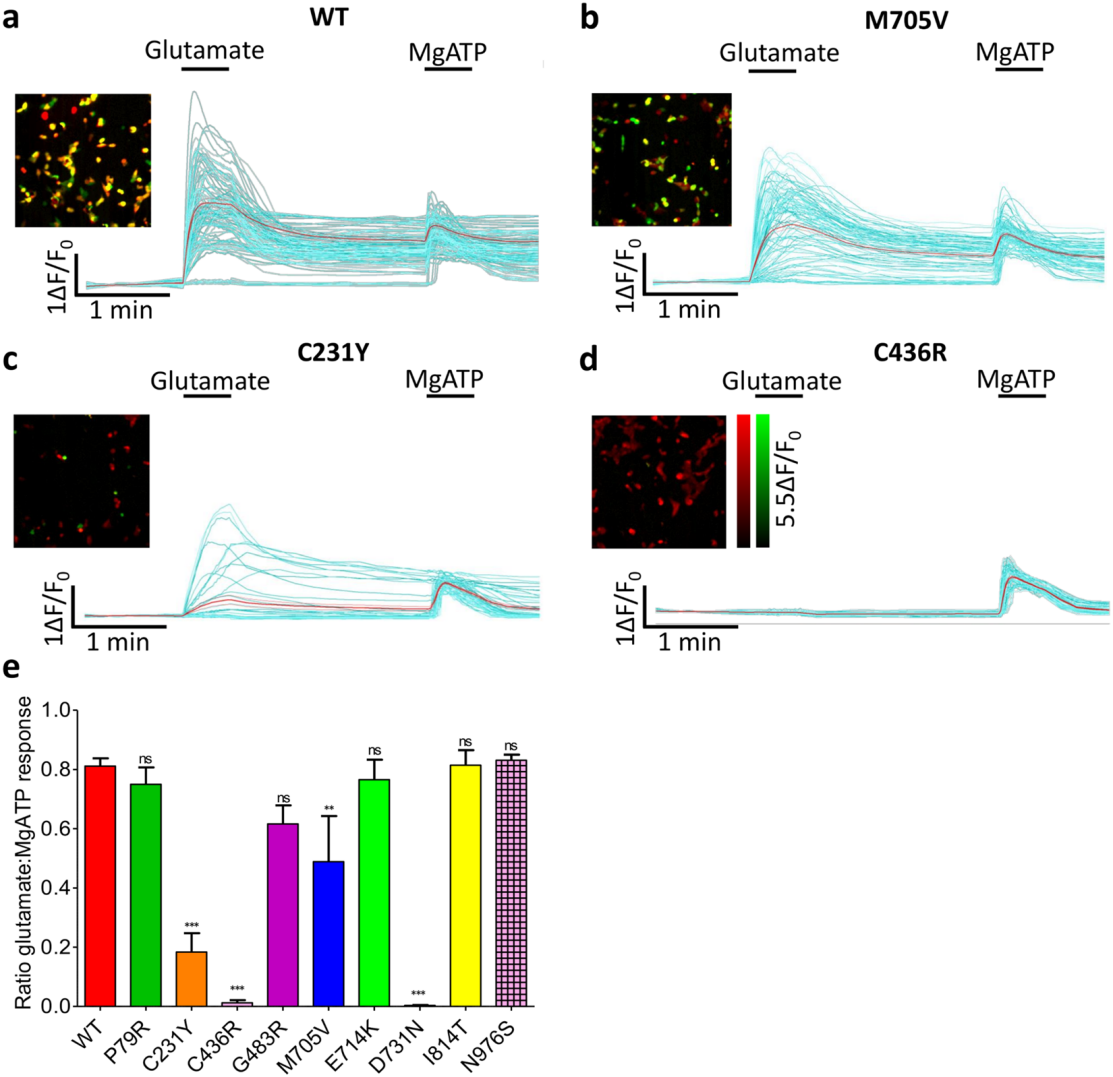
*GRIN2A* mutations alter the number of cells responding to glutamate. (**a–d**) Each panel contains pseudocolour images of HEK cells transfected with WT or mutant *GRIN2A* showing co-localisation of responses to application of 100 µM glutamate +30 µM glycine (green - activation of surface-expressing GluN2A receptors) and subsequently 100 µM MgATP (red - activation of endogenous P2Y receptors). Panels also show single-cell traces from the same experiment, indicating the different effects of these agonists on cells transfected with (**a**) WT, (**b**) M705V, (**c**) C231Y or (**d**) C436R mutants. Individual cell traces displayed in cyan and the mean response shown in red. (**e**) Ratio of the number of transfected HEK cells responding to 100 µM glutamate +30 µM glycine subsequent to 100 µM MgATP. Dunnett’s corrected one-way ANOVA compared to WT, **p < 0.01, ***p < 0.001, ns = non-significant. Data averaged from n = 12 wells, 3 × 10^4^ cells/well, for each construct over 2 assays. Error bars ± SEM.

Ion channel mutations often cause reduced protein expression due to degradation of misfolded proteins within the endoplasmic reticulum (ER) quality control pathway. Using single-cell calcium-flux imaging we investigated if the percentage of HEK cells with NMDARs responding to glutamate changed with mutation type. This was achieved by first perfusing the cells with a maximal glutamate concentration to activate NMDAR expressing cells, and then a maximal MgATP concentration to induce intracellular calcium release via P2Y purinergic receptors found on all HEK cells^24^. Figure 4a show example data for cells transfected with the WT plasmid, where 81% (±2.7% SEM) responded to maximal glutamate. This also shows the transfection efficiency of the plasmids. There was no significant difference for E714K, I814T, D933N and N976S, mutants with no altered glutamate potency. The two mutants who did not respond to glutamate, C436R and D731N, again showed no response here (Dunnett’s corrected one-way ANOVA, F_9,51_ = 23.01, p < 0.001). Interestingly the responses differ for the mutants with decreased glutamate potency, but do not directly correlate with the severity of the agonist potency change; 75% (±5.7%) of P79R cells responded to glutamate (not significant), 61% (±6.3%) of G483R (not significant), 49% (±15%) of M705V (p < 0.01) and 18% (±6.4%) of C231Y (p < 0.001), (Fig. 4b–e), indicating protein degradation and/or trafficking are altered here as well in these mutants.

**Figure 5:**
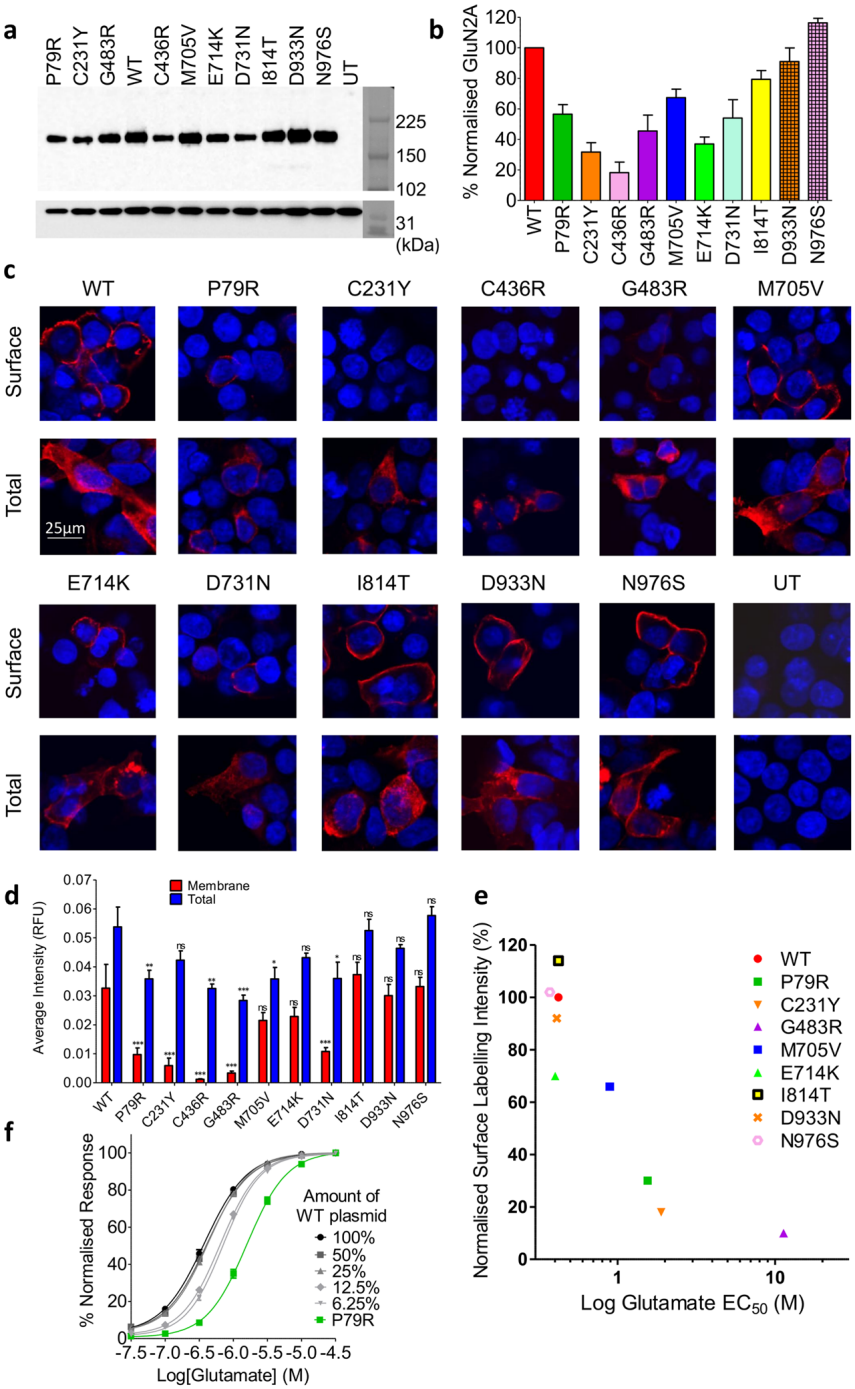
*GRIN2A* mutations reduce total protein levels and membrane trafficking of GluN2A. (**a**) Representative Western blot of HEK lysates probed with anti-GluN2A antibody (top), and anti-GAPDH (bottom) as a loading control. Bands around 180 kDa indicate GluN2A. WT, wild type, UT – untransfected. Right is Amersham Full-Range rainbow molecular weight marker with the blot imaged in visible light. (**b**) Plot of amount of GluN2A protein, normalised to WT, from Western blotting of total cell lysates of transiently co-transfected HEK cells 48-hours post transfection. Average of 3 blots from 3 independent transfections. Error bars indicated SEM. (**c**) Fixed and immunolabelled co-transfected HEK cells with anti-HA antibody (red) to detect surface GluN2A expression, and fixed, permeabilised and immunolabelled with the same antibody to detect total GluN2A protein levels. Scale bar 25 µm. Nuclei stained with Hoechst (blue). (**d**) Quantitation of surface and total GluN2A protein levels, averaged over the total number of cells analyzed for each condition (n is between 903 to 2255 cells), reveals greatly reduced surface expression of GluN2A as measured by fluorescence intensity. Dunnett’s corrected one-way ANOVA for membrane or total intensity as compared to WT *p < 0.05, **p < 0.01, ***p < 0.001, ns = non-significant. Average ± SEM. (**e**) Relative surface levels of NMDARs correlated with the log of glutamate EC_50_ (Pearson’s coefficient of determination r^2^ = 0.77, two-tailed p = 0.002). (**f**) Normalised CRC from single-cell calcium-flux imaging. Response to increasing concentrations of glutamate from HEK cells co-transfected with decreasing quantities of WT *GRIN2A* per well (100% = 320 ng) with standard 320 ng *GRIN1* per well. Response is compared to mutant P79R. Error bars ± SEM.

Western Blotting of total cell lysates from the transfected HEK cells show that GluN2A total protein levels were greatly reduced for most of the mutants, with the lowest relative expression for the two that alter cysteine residues, C231Y (31% ± 6.1) and C436R (18% ± 6.9), (Fig. 5a,b).

### *GRIN2A* mutations can alter GluN2A membrane trafficking

Functional checkpoints in the ER can limit forward trafficking of NMDA receptors to the cell surface^14^. As many mutants reduce total GluN2A protein levels, we wanted to ascertain if they also reduced cell surface expression. Co-transfected HEK cells were fixed with, and without membrane permeabilisation, and incubated with anti-HA-tag antibody, which would ensure binding to the extracellular part of the NMDAR receptor. The average intensity of labelled surface receptor immunofluorescence and of total GluN2A immunofluorescence was calculated for each mutant (Fig. 5c,d)^25^. The immunocytochemistry data explains why cells transfected with mutant C436R do not respond to glutamate – the protein does not traffic to the cell membrane (Dunnett’s corrected one-way ANOVA F_10,55_ = 6.01, p < 0.001). However for D731N some protein does reach the surface of some cells, although is still significantly reduced (p < 0.001), but for this mutant it does not respond to glutamate or glycine. Three of the four mutants with reduced agonist potency, P79R, C231Y and G483R, all have significantly reduced membrane expression (p < 0.001), which is also only present in a very small number of cells. G483R also has significantly reduced total expression, p < 0.001). The reduction of surface expression for M705V is not significant. Interestingly, E714K has a similar level of surface expression to M705V; however, the E714K receptors that reach the cell surface appear to have a normal agonist response, whereas for M705V they do not. Mutants E714K, I814T, D933N and N976S also did not show a significant change in surface or total protein labelling.

The relative level of cell surface expression of the mutant GluN2A constructs correlates well with the glutamate EC_50_ (Fig. 5e). Our results suggest that ligand binding affinity is not determined by cell surface expression. Figure 5f shows that transfection of HEK with 25% less WT *GRIN2A* plasmid compared to the normal 320 ng/well does not alter the response to glutamate. There is a small shift when 12.5% of the plasmid is used, but this does not then shift further at 6.25%, and is much smaller than the significant shift seen for P79R, where the average intensity of surface expression is 30% of WT levels.

### Pharmacological rescue of function can be achieved for selected *GRIN2A* mutants

**Figure 6:**
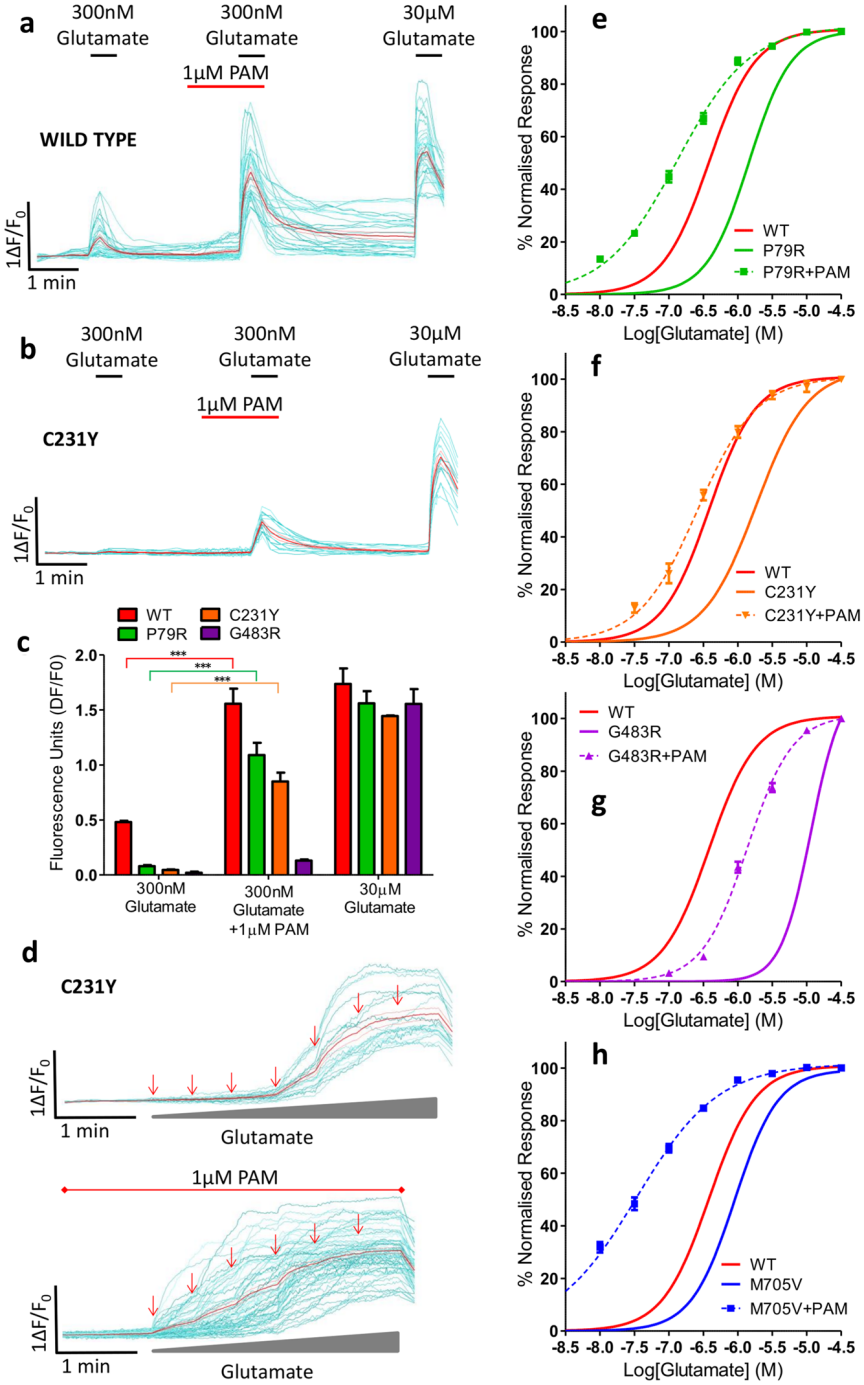
Pharmacological rescue of functional deficits can be achieved for selected *GRIN2A* mutants. Examples of single-cell imaging of HEK transfected with **(a)** WT and **(b)** C231Y mutant showing calcium-influx response to 300 nM glutamate before and after incubation with 1 µM PAM and in comparison to maximal 30 µM glutamate. **(c)** Graph of single cell imaging data showing response of WT and mutants P79R, C231Y and G483R to 300 nM glutamate with and without 1 µM PAM. Bonferroni corrected ANOVA ***p < 0.001, n = 15 wells (over 3 assays) for each construct, 3 × 10^4^ cells/well. Mean ± SEM. **(d)** Examples of single cell imaging of HEK transfected with C231Y mutant showing calcium-influx response to increasing concentrations of glutamate (30 nM, 100 nM, 300 nM, 1 µM, 10 µM and 30 µM arrows) top, and below, increasing concentrations of glutamate (30 nM, 100 nM, 300 nM, 1 µM and 30 µM arrows) with constant 1 µM PAM. Individual cell traces displayed in cyan and the mean response shown in red. **(e–h)** Concentration response curves of mutant (P79R, C231Y, G483R or M705V) *GRIN2A* transfected in HEK cells to increasing concentrations of glutamate (30 nM to 30 µM) during incubation with 1 µM PAM recorded from single-cell calcium-flux imaging. Each graph shows the response of the WT *GRIN2A* construct with no PAM in red (curve from Fig. 3c) and the response of the mutant before (solid line) and after (dashed line) the addition of PAM. p < 0.0001 for LogEC_50_ for each construct + PAM when compared to without PAM addition. Data averaged from n = 12 wells for each construct, 3 × 10^4^ cells/well, (over 3 assays). Error bars ± SEM.

A standard approach for rescuing loss of function phenotypes, as observed here, is the use of pharmacological modulators to enhance receptor function. We hypothesized that mutations which reduce agonist potency could have their function rescued by a selective positive allosteric modulator (PAM). We chose a GluN2A-selective compound recently disclosed in a patent application (compound 275 in ref. 23, GluN2A EC_50_ 12 nM). Initial assays indicated that pre-incubation for 1 minute followed by co-application of 1 µM of the PAM alongside a lower concentration of glutamate significantly increased the calcium influx for the wild type and mutant receptors (Bonferroni corrected one-way ANOVA, F_7,10_ = 55.25, p < 0.001 for WT, P79R and C231Y, G483R is not significant) (Fig. 6a–c and Supplementary Fig. S2). The agonist potency of these mutants was then partially or fully restored to WT levels in the presence of 1 µM PAM followed by sequential addition of increasing concentrations of glutamate (Fig. 6d–h). The EC_50_ for P79R decreased from 1.45 to 0.13 µM (F_1,97_ = 497.5, p < 0.0001) (WT EC_50_ is 0.42 µM), C231Y from 1.89 to 0.26 µM (F_1,77_ = 209.2, p < 0.0001), G483R from 11.30 to 1.37 µM (F_1,87_ = 484.3, p < 0.0001) and M705V from 0.89 to 0.03 µM (F_1,91_ = 500.6, p < 0.0001). The PAM did not have an effect on mutant D731N or C436R (data not shown).

## Discussion

In this study we have carried out a functional analysis of 10 disease-associated missense mutations of *GRIN2A* that were chosen by a bioinformatics and patient-centric approach, and have uncovered a range of molecular consequences. *GRIN2A* mutations in EAS are spread across the gene and our work, combined with others where EAS- or early-onset epileptic encephalopathy associated *GRIN2A* mutations (studied generally in the homozygous state) have been shown to affect zinc sensitivity^16, 26^, increase glutamate potency^27, 28^, alter channel gating kinetics^17, 28^ or eliminate the Mg^2+^ block^11^, indicate a complex pathophysiological picture. Whilst there appears to be a clear rational for alteration of function in some cases, such as substitution of a cysteine residue leading to drastic changes in protein folding and degradation (e.g. C436R here), or substitution of a zinc binding residue slowing zinc dissociation kinetics e.g. R370W in^26^, in other cases further understanding of the structure-function relationships of the NMDAR are needed, such as why L812M in the linker region between the ABD and TM domains enhances agonist potency^28^, whereas I814T studied here, has no effect on agonist potency.

Three mutants have a “double hit” of significantly reduced agonist potency and surface expression; P79R, C231Y and G483R. They were found in children with the most severe EAS subtypes, ECSWS and LKS. M705V also disrupts in a similar manner but to a lesser extent, and is found in a child with less severe Rolandic epilepsy.

Residue G483 occurs on a flexible loop and seems important in the structure of the glutamate binding site, which would be restricted by the change to the large, positive arginine residue. Previously, site directed mutagenesis of the neighboring residue K484 to aspartic acid reduced glutamate affinity 6-fold but had no measurable effect on glycine binding^29^. Interestingly K484 is not conserved in GluN1, whereas G483 is. Residue P79, within the NTD, is conserved across all GluN paralogues and is predicted to make extensive contact with GluN1^30^. Replacement of the proline with the basic arginine residue likely disrupts this contact and the overall NMDAR structure, indirectly affecting the binding of glutamate and glycine. Interestingly Serraz *et al.* also reported that this mutation decreases zinc sensitivity and enhances proton sensitivity, which they hypothesized was also due to the distorted dimer formation between GluN1 and GluN2A^26^. Whilst Serraz *et al.* did not detect protein expression from mutation C231Y over-expressed in oocytes, we detected low levels in transiently transfected HEK-293. We identified a reduction in potency of glutamate and glycine due to the change from cysteine to bulky tyrosine in a hydrophobic cavity of the NTD. This likely causes protein misfolding, leading to an altered binding pocket for agonists and protein degradation. Mutation M705V is located in the ABD, and whilst the substitution is for another hydrophobic residue, this residue is conserved between all GluN subtypes and forms part of the S2 domain of the glutamate binding site (although further away than the other mutations, Fig. 1c).

These four mutations also cause significant reductions in glycine potency. Although glycine binds GluN1, interactions between the ligand binding domains of GluN1 and GluN2A can alter agonist binding potency, as reported for mutations in the ABD of GluN2A^31^. Interestingly, mutation E714K did not appear to alter agonist potency despite its location within the ABD – most likely because the residue is located on the protein’s surface and is not highly conserved. However, this mutation did cause a small, but non-significant reduction in surface expression indicating potential changes in protein folding.

Two mutants create a total lack of NMDAR function as di-heteromeric receptors, C436R and D731N. Mutant C436R is not present at detectable levels on the cell surface and overall GluN2A protein levels are drastically reduced (as also seen in transfected oocytes^26^). It is likely that the replacement of the cysteine residue with polar arginine and breakage of a disulphide bridge causes misfolding and protein degradation for this mutant. This most severe mutation effect is seen in a child with ABPE and occurred *de novo*. On the other hand, mutant D731N does show a low level of surface GluN2A, but shows no response to glutamate or glycine. Interestingly, in studies of the glycine binding site on GluN1, this same residue (D732) when mutated to alanine, caused 90% of the protein to be trapped in the endoplasmic reticulum (ER) upon co-expression with GluN2A and the remainder on the cell surface bound glycine with a 4000-fold reduction in potency^32, 33^. Mutation of D731 to alanine or glutamic acid in GluN2A also produced no response in voltage-clamped oocytes^33^. This residue in GluN2A forms a direct contact with glutamate via a salt bridge, which may indicate why such a severe phenotype was observed.

Surface trafficking of ionotropic glutamate receptors seems to require ligand binding to pass a quality control checkpoint and exit the ER. Indeed the efficiency of glutamate binding to GluN2B mutant subunits correlates with levels of cell-surface receptor and trafficking to synapses. Co-expression with wild-type GluN2B does not enhance surface expression, indicating that occupancy of both glutamate binding sites is required for proper surface delivery^34^. The integrity of the glycine binding site of GluN1 is also required for efficient ER exit and cell surface trafficking, most likely after GluN1-GluN2 subunit assembly^32^. Thus, glycine binding to GluN1, and we hypothesize given the above work, glutamate binding to GluN2A within the ER, controls forward trafficking of the NMDAR to the cell surface. Therefore the patient-derived mutant NMDARs described here, which do not bind these agonists effectively, appear to have reduced or absent trafficking, (Fig. 5e).

Haploinsufficieny of GluN2A subunits through reduction in cell surface levels or lack of response to agonists, as described here, may cause hyper-excitable brain networks through several mechanisms. There may be a relative excess of GluN2B subunit NMDARs in these patients, and with the slower deactivation time-course of GluN2B-containing receptors this could lead to over excitability of the neuronal network^35^. Activity of GluN2A-containing NMDARs is also pivotal in the development and maintenance of the GABAergic function of parvalbumin (PV)-positive interneurons^36^. Thus a reduction in function of GluN2A in these interneurons could lead to a poorly functioning inhibitory network. Expression of GluN2A subunits increases postnatally, replacing GluN2B at the synapse. This results in a balance of synaptic plasticity and stability that is vital for the maturation of associative learning, and dampens hyper-excitation from increased sensory input^37^. A reduction in GluN2A expression during this critical period may create an underdeveloped inhibitory network, again with potential for epileptogenicity.

The current study has some limitations. Missense mutations identified in EAS patients so far are heterozygous, and as the tetrameric NMDAR is formed of two GluN2 subunits and two GluN1, some receptors in the patients will most likely contain one copy of the mutation. We have reported the effects of receptors containing two mutant GluN2A copies, as others have done so, but hypothesize that those with one mutant copy will have an intermediate phenotype for agonist affinity^38^, but remain the same for surface expression^34^. Another limitation is that we have so far only studied the effect of the mutations in a heterologous cell line. HEK-293 are a common and well-validated model for the study of membrane ion channels as they do not contain other channels that need to be blocked, simplifying assay conditions. However, analysis in a more native environment could potentially offer further insights into the effects of the mutations, although they would still need to be overexpressed. We would expect the mutations studied here to have similar effects on NMDAR trafficking and agonist potency in cultured primary neurons, as others have shown translational results^26, 34^. Additional studies will aid clarification of this hypothesis.

We did not find any effects of the mutations I814T, D933N and N976S on the function of NMDARs, however this is not to say that they do not exert an effect on the protein. The C-terminal of GluN2A couples to diverse intracellular cascades such as for scaffolding and post-synaptic signaling, and post-translational modifications are important here^14, 39^. Further work may determine their associations with these residues. Given the low CADD score of N976S, and the fact that it is the only missense variant reported in two unrelated cases to date, it may be that it is a very rare benign variant; however, it has not been reported so far in any control population.

We rescued the function of receptors with reduced glutamate potency by incubation with a novel GluN2A-specific PAM. Due to the differing magnitudes of effect of the mutants on agonist potency, it can be seen that different concentrations of the PAM would be needed for perfect restoration of function. The function of D731N could not be restored by this method, presumably because glutamate does not bind to this mutant. The search for new potentiators for clinical enhancement of NMDAR function has been sporadic. However, with NMDAR hypofunction now shown here to be a disease mechanism in epilepsy, as well as schizophrenia^40^, cognitive decline and Alzheimer’s disease^41^, this may be changing^42^. Rescue of mutant receptors that are trapped within the ER may also be possible using cell-and-ER permeable competitive antagonists, as has been achieved for D732A-GluN1 using the competitive glycine site antagonist DCKA^32^, however cell permeability may be an issue for GluN2A-specific antagonists.

The mutations studied here show a range of phenotypic deficits, and combined with the work of others indicate a complex picture of molecular consequences. Modern translational medicine techniques will be needed to identify patient mutations through high-throughput sequencing, and confirm the type of molecular dysfunction before the identification of potential therapeutic compounds *in vitro*. This strategy of personalized medicine may allow for a repositioning of certain approved-compounds and a reduction in the number of antiepileptic medications used, and could dramatically alter disease progression and the development of the affected child.

## Methods

### Selection of mutants and functional hypotheses

31 missense mutations from individuals with EAS disorders^15–17^ were assigned Combined Annotation Dependent Depletion (CADD) scores^20^ using the web tool http://cadd.gs.washington.edu/ v1.3 to create predictions of deleteriousness. These CADD scores were compared to those for controls from the Exome Variant Server (EVS) (http://evs.gs.washington.edu/EVS/ assessed 05/2016) where 65/6474 individuals sequenced across *GRIN2A* had a missense variant. A scaled CADD score cut off of 20 was used to indicate a variant as highly likely to be deleterious.

A contribution to the selection of the mutants included here was a hypothesis of the function of each mutant based on their location in available structures of GluN2A (or homologous GluN2B structures). P79R is present in the NTD, this mutation was hypothesised to affect the conformation of that region of the protein, in addition to affecting the contacts that the proline residue has with GluN1. C231Y is also present in the NTD, this mutation causes a significant change in residue size and was hypothesised to affect the conformation of this region of the protein. C436R is present in the ABD and is involved in a disulfide bond; this mutation was hypothesised to be highly destabilizing. G483R is present in the ABD in close proximity to the glutamate binding site and thus predicted to affect binding affinity. M705V is present in the ABD, albeit slightly further away from the glutamate binding site; some protein conformational change was hypothesised. E714K is present in the ABD, but on the protein surface. In some crystal structures it is not full resolved suggesting it is flexible and non-essential for protein stability, thus a limited effect on glutamate binding was hypothesised. D731N is present in the ABD and forms a direct contact to glutamate via a salt bridge; this mutation was hypothesised to have a significant effect on glutamate binding although the protein was expected to still be able to fold correctly. I814T is present on the linker between the ABD and TMD, the effect of this mutation was unclear from a structural perspective. Mutations D933N and N976S are both present in the CTD and are not structurally characterized so hypotheses could not be generated.

### DNA constructs

Human *GRIN2A* (GenBank accession: NM_000833.3) cDNA clone was purchased from OriGene Technologies (Cat#: SC122120). The various mutants of *GRIN2A* and insertion of the HA-tag into *GRIN2A* were generated by PCR-based mutagenesis using the wild-type cDNA clone as template. Mutations inserted were: P79R, C231Y, C436R, G483R, M705V, E714K, D731N, I814T, D933N and N976S. The nucleotide sequences encoding full-length wild-type and various mutants were inserted into pcDNA3.1 (+) (Invitrogen) with the cytomegalovirus (CMV) enhancer-promoter for high expression, and verified by DNA sequencing. Human *GRIN1* (GenBank accession NM_000832.5) cDNA clone was also purchased from Origene Technologies (Cat#: SC308819) and inserted into a second pcDNA3.1 (+) vector. An empty pcDNA3.1 (+) vector was used as a control in the live cell imaging assays and co-transfected with the *GRIN1* plasmid. Transfection efficiency was assessed in the calcium flux assay as the number of HEK cells responding to maximal glutamate (Fig. 4e) vs. those responding to MgATP, as NMDAR can only function if both *GRIN1* and *GRIN2A* plasmids transfect and express the protein.

### Cell culture and transient transfection

HEK-293 cells were cultured in high glucose DMEM medium (Gibco) containing 2 mM penicillin/streptomycin and glutamine (Gibco) and 5% bovine calf serum (Gibco). Cells were plated at a density of 3 × 10^4^ cells/well in 96-well black-walled poly(D)-lysine coated plates (Corning) with the addition of 1mM Ketamine (Sigma) in the culture media. Cells were transfected using 0.4 µl Lipofectamine 2000 (ThermoFisher) per well, with 320 ng *GRIN1* construct and 320 ng of a *GRIN2A* construct. Transfection with *GRIN1* is presumed in the figure legends. For the transfection assay (Fig. 5f), varying amounts of WT *GRIN2A* plasmid (320, 160, 80, 40 and 20 ng) were added per well, along with 320 ng *GRIN1*. Cells were kept in an incubator at 37 °C and 5% CO_2_ and used for all assays 48-hours after plating and transfection.

### Continuous live cell imaging – cell toxicity assay

HEK-293 cells were seeded and transiently transfected as detailed above, but without Ketamine in the culture media. Cytotox-Red dye (Essen BioScience) was added to each well to 1:400 dilution for labelling of nucleic acids in dead cells upon excitation at 585 nm. Plates were imaged one field per well every 6 hours in the Incucyte ZOOM live cell imaging system (Essen BioScience) for 48 hours. Images were analysed with the Incucyte ZOOM software and data normalised to the initial confluencey of cells in each well to control for inter-well plating variability.

### Calcium flux assays

HEK-293 cells were seeded and transiently transfected as detailed above. After 48 hours, media was removed and 100 µl of buffer containing the cell-permeant calcium binding dye at 4 µM, Fluo-4 AM (ThermoFisher), 0.1% pluronic acid (ThermoFisher) and 1mM Ketamine was added per well. Details of the buffers and method used for the single cell calcium flux imaging are as in^22^. A modified HEPES-buffered Tyrode’s solution (HBTS) (Invitrogen) was used throughout the experiments and called “perfusion buffer”, containing (mM): 135 NaCl, 5 KCl_,_ 2.5 CaCl_2_, 10 HEPES, 10 glucose, pH 7.2). Transfected cells were identified as responding to compound addition due to fluorescence of the Fluo-4 AM dye upon calcium influx into the cells. Data were analysed by averaging individual traces collected from cells in multiple wells of the 96-well plate. N = well number, and number of experiments is listed below each figure. Nonlinear regression using the least squares fit of the log(agonist) vs. response – variable slope (four parameters) was applied to the fluorescence intensity values with background removed, and were normalised to the maximal response for each mutant.

For all assays described below, compounds are diluted in perfusion buffer and glycine (Tocris) is at 30 µM unless otherwise stated. For the glutamate CRC assays, increasing concentrations of glutamate (ranging from 30 nM to 30 µM) (Sigma) were perfused over the cells every 30 seconds, whilst keeping constant 30 µM glycine. For the glycine CRC assays, increasing concentrations of glycine from 30 nM to 30 µM were perfused over the cells, with constant 3 µM glutamate. For the cell counting assay, initially 100 µM glutamate with 30 µM glycine were perfused over the cells for 30 seconds, followed by a wash for 3 minutes and then perfusion with 100 µM MgATP (Sigma) for 30 seconds. The GluN2A-selective PAM was synthesized in-house following the structure of the compound named “example 275” from the referenced patent application^23^. The structure of the compound is listed in the patent, and the potency for GluN2A is listed with an EC_50_ of 12 nM. This compound is subsequently referred to as the PAM. For the assays using the PAM, two different protocols were used, as described in Fig. 6.

### Patch Clamp Recordings

Whole–cell voltage clamp recordings were carried out on transfected HEK-293 cells at a holding potential of −60 mV (at 21 °C) using an Axopatch 200A amplifier (Molecular Devices). Current data were recorded at 10 kHz using a DA/AD interface (Digidata 1322A, Molecular Devices). Patch pipettes were pulled using a horizontal puller (Sutter Instruments) from thin-walled borosilicate glass (Harvard apparatus, Ltd.) that gave resistances between 4 and 7 MΩ when filled with the intracellular electrode solution. The pipette solution comprised of (in mM) 140 K-Gluconate, 1 MgCl_2_, 4 MgATP, 0.5 EGTA, 10 HEPES, (adjusted to pH 7.3 with KOH, all Sigma). Cells were perfused with the same perfusion buffer used in the calcium flux assay containing 30 µM glycine, and glutamate from 30 nM to 30 µM as detailed in Fig. 3, using a multichannel perfusion system (Model BPS-8, Scientifica). Transfected cells were identified by an initial large response to maximal glutamate and glycine and used for subsequent recording protocols.

### Western Blotting

HEK-293 cells were harvested 48-hours after transient transfection. Proteins were extracted using RIPA buffer (Sigma) with Halt phosphatase inhibitor (ThermoFisher) and protease inhibitor cocktail (Calbiochem) for 10 minutes on ice. Proteins (4 µg/well) were prepared in 1xLaemmli buffer (Bio-Rad) and 5% β-mercaptoethanol (Sigma), heated at 37 °C for 15 min and resolved on a 3–8% Tris-acetate NuPAGE Novex gel (ThermoFisher). Proteins were then transferred onto Hybond-ECL nitrocellulose membrane (Amersham) which was cut on the 38 kDa marker and the top part (>38 kDa) incubated with anti-GluN2A antibody (M264, Sigma) at 1:1000 and the bottom part with anti-GAPDH antibody (AM4300, Ambion) 1:10,000. Primary antibodies were detected with horseradish peroxidase conjugated anti-rabbit (GluN2A) and anti-mouse (GAPDH) antibodies (Sigma) at 1:10,000 dilution, followed by signal development with SuperSignal West Dura reagent (ThermoFisher). Protein bands were detected on an Image Quant Las 400 Mini apparatus (GE Healthcare). Images were also obtained in the visible light spectrum for alignment of the molecular weight marker bands. Values reported are means of four different transfection, lysis and Western blotting experiments, i.e. N = 4.

### Immunocytochemistry

48-hours after transfection HEK cells were treated in two different ways: 1) For total GluN2A labelling, cells were fixed with 4% paraformaldehyde for 10 min before blocking/permeabilisation with 5% BSA (Thermo Fisher) 0.1% Triton X-100 (Sigma) in PBS for 30 min at room temperature. 2) For membrane-only staining, cells were blocked in PBS with 5% BSA and 1mM Ketamine for 1 hour at room temperature. For both methods, HA-tagged GluN2A was immunolabelled using anti-HA antibody (H6908) at 5 µg/ml in PBS with 0.5% BSA, (and 0.1% Triton X-100 for the total GluN2A labelled cells), for 2 hours at 4 °C, followed by washing. The membrane-only cells were now fixed with 4% paraformaldehyde. Both cell types were then incubated for 1.5 hours with Alexa Fluor 555-conjugated donkey-anti-rabbit secondary antibody at 1:1000 in the dark (A-31572, Thermo Fisher). Cells were finally then incubated for 10 min at room temperature with Hoechst 33342 nuclear stain (Thermo Fisher). Stained cells were viewed by confocal microscopy using the Olympus FV1000. Image files were analysed by using Cell Profiler^24^. Briefly images were segmented by using the nuclear staining channel followed by measurement of average fluorescence intensity in the Alexa-555 channel.

### Statistical Analysis

Data were analysed using Prism 5 (Graph Pad). Where appropriate, data were tested for normal distribution using the Kolmogorov-Smirnov test and passed. Alpha was 0.05. Analysis of statistical significance was assessed using either a one way ANOVA corrected for multiple testing with Dunnett’s correction for Figs 2c, 4e and 5d, and with Bonferroni correction for 6c, and the extra-sum-of-squares F-test of best-fit values of LogEC_50_ used for Figs 3c–f, 5f and 6e–h. Correlation between surface levels of Glu2NA expression and EC_50_ for the mutations was assessed using Pearson’s coefficient of determination in Fig. 5e.

## Electronic supplementary material

Supplementary Figure S1 and S2
