# Supplementary material for: Epilepsy-associated GRIN2A mutations reduce NMDA receptor trafficking and agonist potency – molecular profiling and functional rescue
Source: Sci Rep. 2017 Feb 27;7:66. doi: 10.1038/s41598-017-00115-w (PMC5427847; doi:10.1038/s41598-017-00115-w)
Supplement: Supplementary file 1 — Supplementary Figure S1 and S2 [file 41598_2017_115_MOESM1_ESM.pdf]

Epilepsy-associated *GRIN2A* mutations reduce NMDA receptor trafficking and agonist potency – molecular profiling and functional rescue

L. Addis D.Phil[1, 2]*, J.K. Virdee PhD[2], L.R. Vidler PhD[2], D.A. Collier PhD[2], D.K. Pal MD, PhD[1] and D. Ursu PhD[2]

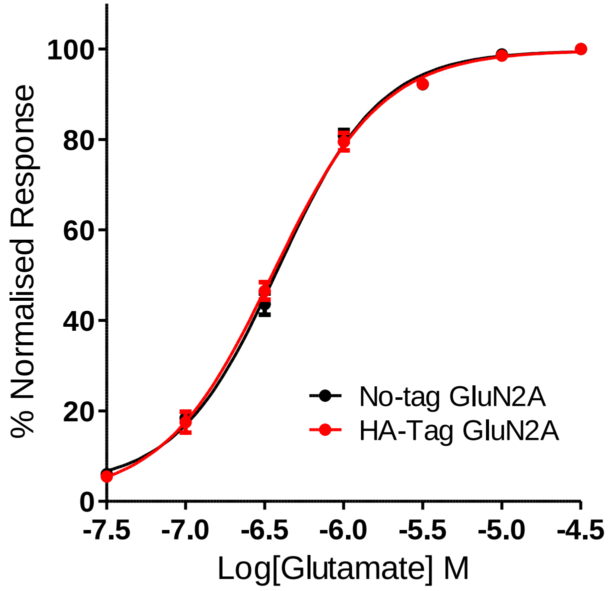

Supplementary Figure S1
Normalised concentration response curves (CRCs) to increasing concentrations of glutamate from single cell calcium-flux imaging of HEK cells transiently co-transfected with *GRIN1* and HA-tagged *GRIN2A*, or *GRIN1* and no-tag *GRIN2A*. Graphs show there is no difference in the response to glutamate when the HA-tag is added to the plasmid.

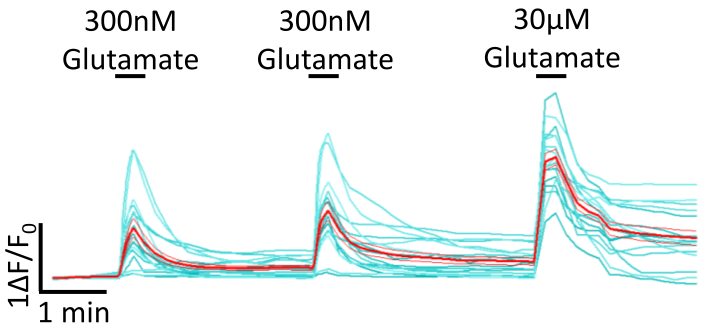

Supplementary Figure S2
Example of single cell calcium imaging traces of HEK transfected with *GRIN1* and WT *GRIN2A* plasmids responding to repeated addition of 300nM glutamate, followed by 30µM Glutamate. There is no significant difference between the repeated additions of 300nM glutamate. Individual cell traces displayed in cyan and the mean response shown in red
